# Kinetics of adrenomedullin pathway activation in a porcine sepsis model and a human cohort of sepsis and septic shock

**DOI:** 10.1038/s41598-025-19278-y

**Published:** 2025-09-24

**Authors:** Christoph Thiele, Yulia Ilina, Paul Kaufmann, Gero Springsfeld, Michaela Press, Oliver Hartmann, Andreas Bergmann, Thomas Breuer, Gernot Marx, Tim-Philip Simon

**Affiliations:** 1 Department of Intensive Care and Intermediate Care, University Hospital RWTH Aachen, Pauwelsstr. 30, 52074 Aachen, Germany; 2 PAM Theragnostics GmbH, Neuendorfstr. 15A, 16761 Hennigsdorf, Germany; 3 Sphingotec GmbH, 16761 Hennigsdorf, Germany

**Keywords:** Sepsis, Septic shock, Adrenomedullin (ADM), Peptidylglycine α-amidating monooxygenase (PAM), AdrenOSS-1, Biomarker, Diagnostic markers, Predictive markers, Prognostic markers

## Abstract

Sepsis is a life-threatening condition characterized by endothelial dysfunction. The peptide hormone adrenomedullin (ADM) plays a key role in sepsis owing to its potent vasodilatory effects, ability to maintain vascular integrity, and critical role in modulating immune responses and reducing inflammation. To gain its biological activity, the inactive ADM precursor (ADM-Gly) is converted into its active form (bio-ADM) by peptidylglycine α-amidating monooxygenase (PAM). Here, we present hourly resolved kinetics of ADM activation during early sepsis onset in a porcine model and analyze the AdrenOSS-1 human cohort data to assess biomarker changes in advanced sepsis progression. The porcine model data showed that both bio-ADM and ADM-Gly mean concentrations rose within the first two hours post-induction, preceding measurable sepsis onset, with a greater increase in ADM-Gly (260.8 ± 92.0 pg/mL) compared to bio-ADM (28.0 ± 12.9 pg/mL). PAM activity increased at 6 h (39.3 ± 10.5 Units), accompanied by a rise in the bio-ADM/ADM-Gly ratio. AdrenOSS-1 study revealed that ICU sepsis patients had higher ADM-Gly (121.5 pg/mL [IQR: 44.4–284.1]) and PAM activity (23.5 Units [IQR: 17.7–32.7]) than controls. Elevated ADM-Gly (> 730 pg/mL) and PAM activity (> 35.1 Units) were associated with increased 28-day mortality, with non-survivors exhibiting higher ADM-Gly (603.5 pg/mL [IQR: 131.1–1443]) and PAM activity (28.3 Units [IQR: 19.0–45.1]) than survivors. This study provides novel insights into the dynamics of adrenomedullin (ADM) homeostasis during sepsis progression, highlighting the critical interplay between its glycine-extended precursor (ADM-Gly), fully active form (bio-ADM), and the amidating enzyme PAM. The findings demonstrate that early and significant elevations in ADM-Gly, accompanied by delayed PAM activity, result in incomplete ADM amidation, compromising endothelial barrier function. Elevated ADM-Gly and PAM activity were associated with increased sepsis severity and 28-day mortality, while a higher bio-ADM/ADM-Gly ratio was linked to improved survival. These results underscore the potential of ADM-Gly, bio-ADM, and PAM as biomarkers for sepsis severity and prognosis, and support therapeutic strategies aimed at enhancing PAM activity to restore endothelial integrity and improve patient outcomes in sepsis.

## Introduction

Sepsis is a well-known contributor to mortality worldwide. The exact number of cases is difficult to assess; however, a study in 2020 estimated 48.9 million cases and 11 million sepsis-related deaths worldwide in 2017^1^. Sepsis is characterized by life-threatening organ dysfunction, possibly leading to septic shock, multiple organ failure, and death owing to a dysregulated host response to pathogen infection^2^. Multiple processes, including activation of inflammatory pathways and compromised vascular tone and integrity, are involved in the pathogenesis of sepsis and septic shock, resulting in endothelial dysfunction^3^.

Vascular function is essential for survival in septic shock. The functional vascular endothelium plays a pivotal role in the regulation of molecule and substrate diffusion between intravascular and interstitial spaces, as well as in vessel tone and angiogenesis. Physiologically, under inflammatory conditions, the leakage of fluids and inflammatory substances, as well as leukocytes into the interstitium, plays an important role in combating local infections in tissues. However, this process is exacerbated by sepsis. The endothelial cell-cell junction is disrupted due to disturbed endothelial cell signaling and death, resulting in loss of the endothelial cell barrier. This leads to excessive fluid leakage, which subsequently reduces blood pressure and causes tissue hypoperfusion. The fluid accumulates, interstitial edema occurs, and shock develops^3–6^.

The ubiquitously expressed peptide hormone adrenomedullin (ADM) has emerged as a central player in sepsis, owing to its vasodilatory properties and role in preserving endothelial barrier function. ADM is released in response to compromised endothelial integrity, inflammatory stimuli and hypoxia, acting as both a biomarker and a mediator of sepsis^7^. The full biological activity of ADM is achieved in the final step of C-terminal amidation of its precursor, glycine-extended ADM (ADM-Gly), which is exclusively catalyzed by peptidylglycine α-amidating monooxygenase (PAM)^8,9^. This final step results in a fully biologically active 52-amino acid peptide bio-adrenomedullin (bio-ADM).

The vasodilatory effect of bio-ADM is exerted by binding to CRLR + RAMP2/3 receptors located in both endothelial and vascular smooth muscle cells (VSMCs)^10^. This leads to two distinct signaling pathways. The first is a direct pathway via VSMCs, which triggers an increase in cyclic adenosine monophosphate (cAMP) levels upon binding of bio-ADM to the receptor. The resulting relaxation of the smooth muscle cells causes vasodilation. Similarly, the indirect endothelial-dependent pathway induces local release of nitric oxide (NO), which leads to vasorelaxation^10,11^.

In addition to its vasodilatory properties, bio-ADM plays a crucial role in maintaining endothelial barrier integrity through cAMP, cGMP and intracellular Ca2 + signaling^12,13^ Bio-ADM has been demonstrated to reduce the effects of thrombin and peroxide through cAMP-dependent relaxation of the microfilament system counteracting the phosphorylation of myosin light chains^14^. Furthermore, bio-ADM stabilizes the lymphatic endothelial barrier by reorganizing tight junction and adherens proteins^15^, promotes angiogenesis^16^, and inhibits endothelial cell apoptosis through nitric oxide production^17^. It also prevents the formation of stress fibers and gaps between cells in the layers of both human umbilical vein endothelial cells and porcine pulmonary artery endothelial cells^18^.

In patients with sepsis, prompt diagnosis and treatment are vital as every minute is critical. Understanding the early stages of sepsis pathogenesis is essential to prevent delays in therapy and to ensure targeted treatment. However, most studies have focused on patient parameters after ICU admission, highlighting the importance of developing sepsis models that can capture the initial stages of the disease.

ADM shows promise as a therapeutic target in patients with sepsis. However, our understanding of how ADM activation is regulated in the bloodstream during the early stages is limited, particularly regarding the role of PAM in maintaining ADM homeostasis.

To address these gaps, we investigated the course of sepsis by analyzing changes in the ADM amidation state using two different strategies. First, we utilized a septic porcine model to monitor the bi-hourly changes in the concentration of bio-ADM, ADM-Gly, and PAM activity during the early stage of sepsis, which started from sepsis induction to 14 h afterwards. Second, we analyzed the same parameters in a human septic cohort, the multinational Adrenomedullin and Outcome in Sepsis and Septic Shock 1 (AdrenOSS-1) study, in plasma samples taken after ICU admission, where the stage of sepsis was already advanced. This approach provided us with a comprehensive insight into the course of sepsis, starting from timepoint zero (ICU admission) to 28 days after sepsis diagnosis, with respect to peptide hormone homeostasis and PAM activity.

## Materials and methods

### Animals

We anesthetized and ventilated nine female German Landrace pigs (33 ± 1.5 kg body weight (BW) (mean ± standard deviation (SD)) and followed the standard procedures for laboratory animal care. The institutional and local committee on the care and use of animals (Landesamt für Natur, Umwelt und Verbraucherschutz Nordrhein-Westfalen, Germany, AZ 84-02.04.2016.A194) approved this study. The animal experiments were conducted in a university animal research facility (Institute for Laboratory Animal Science, RWTH Aachen University).

All methods were performed in accordance with the relevant guidelines and regulations. We received and purchased the animals from the Institute of Laboratory Animal Science. The animals came from a regional pig breeder (Heinsberg, Germany). The study is reported in accordance with ARRIVE guidelines.

### General anesthesia and catheterization

Animals were premedicated with azaperone (1–2 mg/kg BW) and ketamine (10 mg/kg BW), and general anesthesia was induced by intravenous injection of propofol (1–2 mg/kg BW). The animals were orally intubated and placed in the supine position. General anesthesia was maintained with an infusion of propofol (5–10 mg/kg BW/h) and fentanyl (4–10 µg/kg BW/h). Controlled pressure mode ventilation was chosen to ventilate the animals with an inspiratory oxygen fraction of 0.5, inspiratory/expiratory ratio of 1:1.5, PEEP of 5 cm H_2_O, and tidal volume of 8–10 ml/kg BW. The respiratory rate was set to maintain PaCO_2_ of 4.7–5.9 kPa. The body core temperature was maintained at a minimum of 37.5 °C with a warming blanket. A central venous catheter (Arrow International, Inc., PA, USA) was inserted into the external jugular vein and a PiCCO^®^ (Pulse index Contour Cardiac Output) arterial thermodilution catheter (PULSION Medical Systems, Feldkirchen, Germany) was inserted into the femoral artery by transcutaneous puncture. At the end of the study, the animals were euthanized in the presence of a veterinarian with a lethal dose (80–160 mg/kg BW) of pentobarbital (Narcoren^®^, Merial, Hallbergmoos, Germany) while they were still under deep anesthesia.

### Escherichia coli fibrin clot

In this model, we used an *E. coli*-laden clot with 7–9 × 10^11^ colony-forming units (CFUs) per kg/BW to induce septic shock. The clot consisted of a sterile solution of porcine fibrinogen (Sigma-Aldrich Inc., St. Louis, MO, USA) (the final volume was adjusted to the pig’s weight) and *E. coli*, to which thrombin from bovine plasma was added. The clots were incubated for 30 min at room temperature.

### Experimental protocol

**Fig. 1:**
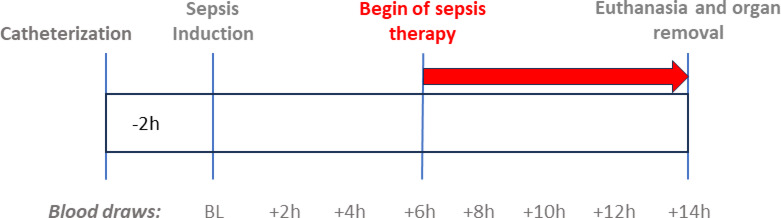
Schematic representation of a porcine septic shock model.

During catheterization, the animals received 10 ml/kg BW/h of balanced crystalloid solution (Sterofundin Iso, B. Braun, Melsungen, Germany). Sepsis was induced by placing an *E. coli*-laden clot in the abdominal cavity. Six hours after sepsis induction, therapy for septic shock started using balanced crystalloids and noradrenaline titrated to maintain a MAP > 65 mmHg and a stroke volume variation < 12%. Sepsis therapy was continued for an additional 8 h. Blood samples were collected before sepsis induction (baseline (BL)) and at 2, 4, 6, 8, 10, 12, and 14 h after sepsis induction. A schematic of the experimental setup is shown in Fig. 1.

### Human cohort

ADM-Gly concentrations together with PAM activity (PAM-AMA) were measured in a randomly chosen cohort of 199 individuals from the prospective observational multinational Adrenomedullin and Outcome in Sepsis and Septic Shock 1 study (AdrenOSS-1). A detailed description of this cohort has been presented elsewhere^19^. The study protocol was approved by the local ethics committees, and the study was conducted in accordance with Directive 2001/20/EC as well as good clinical practice (International Conference on Harmonization Harmonized Tripartite Guideline version 4 of May 1, 1996, and decision of November 24, 2006) and the Declaration of Helsinki. Patients were included from June 2015 to May 2016. Informed consent was obtained from all patients or their lawful representatives prior to enrollment in the study. Patients were treated according to local practice, and treatments as well as procedures were registered.

Briefly, the term “sepsis” refers to the definition of Sepsis-3, as described by Singler et al. The term ‘septic shock’ was based on the definition provided by Levy et al.^2,20^. Two independent cohorts comprising 98 and 120 self-reported healthy individuals were used as control groups.

### Biomarker analysis

Bio-ADM concentration was determined using sandwich chemiluminescence immunoassays developed by SphingoTec GmbH^21^ and previously reported by Mebazaa et al. 2018^19^. The concentration of ADM-Gly was measured in a one-step high-throughput microtiter plate-based chemiluminescence immunometric assay with solid phase and tracer antibodies specifically directed against the middle and C-terminal regions of ADM-Gly, respectively^22^. Amidating activity of the PAM enzyme was determined in an amidation assay using synthetic ADMGly as a substrate, as described elsewhere^23^. The measured activity was expressed in AMA-units, with one AMA-unit equivalent to the generation of 1 µg of bio-ADM in 1 L of the sample over a period of 1 h.

### Statistical analysis

All statistical analyses were carried out with GraphPad Prism 10.4.1 (627).

In the porcine model, biomarker dynamics were analyzed using two-way ANOVA on log-transformed data to ensure normality. Comparisons were made relative to baseline (t = 0), with Dunn’s correction applied for multiple comparisons. Longitudinal changes in physiological and biochemical parameters between baseline and 8 h post-sepsis induction were assessed using two-tailed paired t-tests or one-way ANOVA to account for inter-individual variability.

For the human AdrenOSS-1 prospective cohort, comparative analysis of ADM-Gly and PAM activity across groups (sepsis, septic shock, and healthy controls) was performed using one-way ANOVA with Dunn’s correction. Differences in bio-ADM/ADM-Gly ratios between survivors and non-survivors were evaluated using nonparametric Mann-Whitney U tests.

Survival analysis was conducted to assess 28-day mortality in relation to PAM activity and ADM-Gly levels using the log-rank Mantel-Cox test, with hazard ratios calculated via the Mantel-Haenszel method. Patients were stratified by predefined biomarker thresholds (ADM-Gly = 730 pg/mL and PAM activity = 35.1 Units). Kaplan-Meier survival curves based on these cut-off values were generated for illustrative purposes.

Descriptive statistics were used to summarize data, with results reported as mean ± standard deviation (SD) for normally distributed variables and as medians with interquartile ranges (IQRs) for non-normal distributions.

## Results

**Fig. 2:**
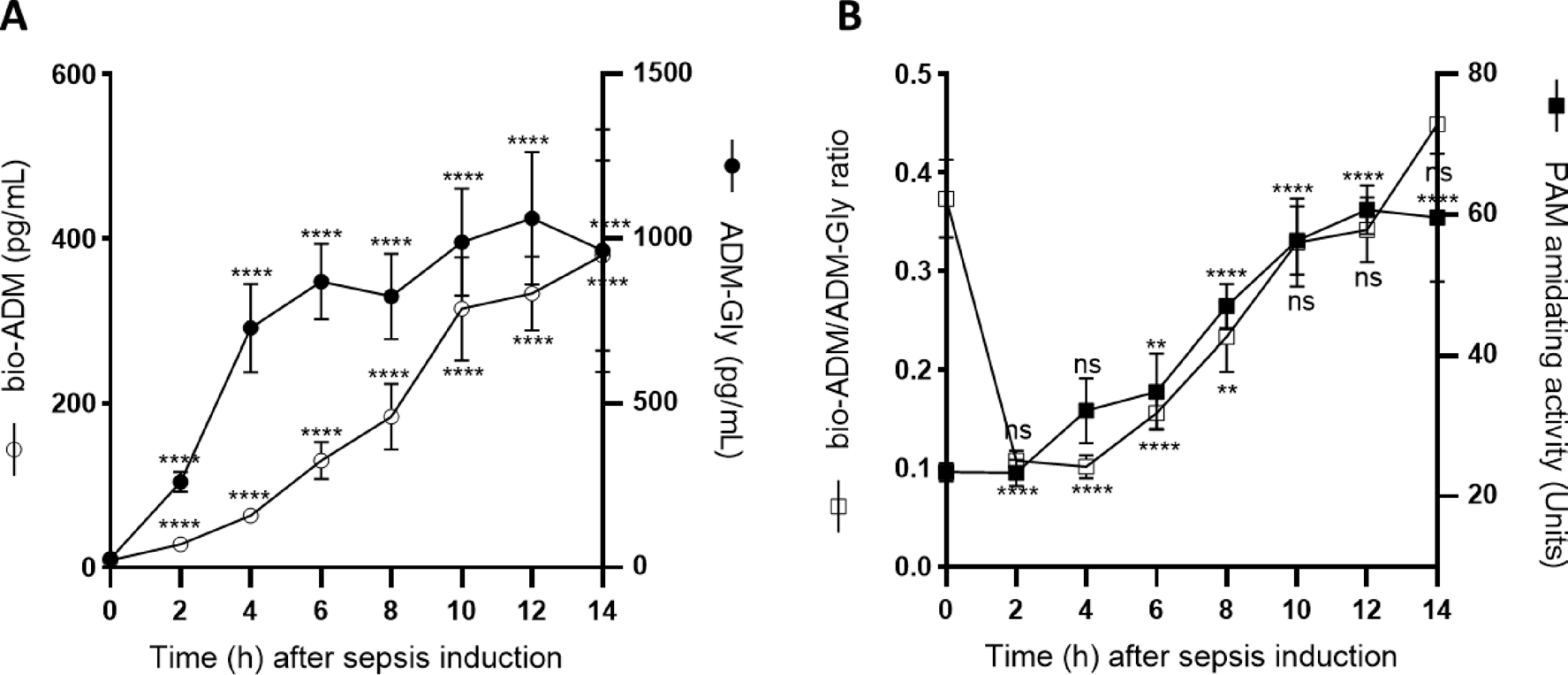
Time-dependent kinetics of biomarkers ADM-Gly and bio-ADM (**A**), bio-ADM/ADM-Gly ratio and PAM amidating activity (AMA) (**B**) after induction of a septic shock in a porcine model. ADM-Gly (black circles) and bio-ADM (open circles) are shown as pg/mL. PAM-AMA (black squares) is shown as AMA units (µg/(L*h). The bio-ADM/ADM-Gly ratio (open squares) was calculated as bio-ADM/ADM-Gly using the concentrations of both peptides in pg/mL. Significance was assessed using log-transformed data with t = 0 as the reference point via a two-way ANOVA, using Dunn’s correction for multiple comparisons. p-values < 0.05 were considered significant. **: *p* ≤ 0.096; ****: *p* < 0.0001; n.s.: not significant. Animal survival: Of nine animals, no mortality was observed from 0 h to 8 h post-induction. At 10 h, two animals had died; by 12 h, three animals in total; and by 14 h, six animals.

The results of the porcine sepsis model showed a rapid significant increase in the basal ADM-Gly mean concentration (± SD) from 26.5 ± 11.2 pg/mL to 728.7 ± 378.3 pg/mL within the first four hours after the induction of sepsis, which was followed by a plateau for the next ten hours (Fig. 2A, Tab. S1). Bio-ADM showed a slower increase, reaching a concentration of 63.4 ± 20.5 pg/mL within four hours and ultimately plateauing at 314.5 ± 165.6 pg/mL ten hours after sepsis induction (Fig. 2A). The basal bio-ADM concentration was 8.8 ± 1.3 pg/mL. The first significant elevation in the amidating activity of 39.3 ± 10.5 Units was measured six hours after the induction of sepsis, when compared to the basal amidating activity of 23.4 ± 3.9 Units (Fig. 2B). This increase coincided with the increase in the bio-ADM/ADM-Gly ratio (0.10 ± 0.03 at 4 h to 0.16 ± 0.05 at 6 h, Fig. 2B, Tab. S1). All animals reached 8 h after sepsis induction. Two died before 10 h (remaining *n* = 7), one before 12 h (remaining *n* = 6), and three died before 14 h, the point of euthanasia and organ removal (remaining *n* = 3). Table 1 shows the changes in hemodynamic, respiratory, hematological, biomarker, metabolic, and fluid/electrolyte parameters in sepsis-induced animals at baseline and at 8 h post-sepsis induction. These changes demonstrate the onset and full manifestation of sepsis, as indicated by significant alterations in vital signs, biomarkers, and hematological values. The comprehensive bi-hourly time course of all parameter changes is presented in Table S1. Dipeptidyl peptidase 3 (DPP3) is an aminopeptidase that is involved in the development of circulatory shock^24,25^. In an experimental septic shock model, the inhibition of circulating DPP3 by an anti-DPP3 antibody reduced catecholamine exposure and myocardial injury. In the present model, however, DPP3 increases with disease evolution were not observed. This finding demonstrates that, among the multiple processes implicated in the pathogenesis of sepsis, the endothelial-driven pathway is predominant in this animal model.

**Table 1:** Time course of physiological and biochemical parameters in Porcine sepsis-induced model. DPP3 (Dipeptidyl peptidase 3), CO (Cardiac Output), HR (Heart Rate),* MAP (Mean Arterial Pressure)*, SVR (Systemic Vascular Resistance), EVLW (Extravascular Lung Water), gapCO2 (Carbon Dioxide Gap), mPAP (Mean Pulmonary Artery Pressure), SvO2 (Mixed Venous Oxygen Saturation), Hb (Hemoglobin), SaO2 (Arterial Oxygen Saturation), Hct (Hematocrit), cLac (Lactate Concentration), ScvO2 (Central Venous Oxygen Saturation), FiO2 (Fraction of Inspired Oxygen), SpO2 (Peripheral Oxygen Saturation), ALP (Alkaline Phosphatase), AST (Aspartate Aminotransferase), ALT (Alanine Aminotransferase), GGT (Gamma-Glutamyl Transferase), CRP (C-Reactive Protein), IL-6 (Interleukin 6), TNF (Tumor Necrosis Factor). The values are presented as mean ± standard deviation for *n* = 9 animals in each group, at baseline (BL) and 8 h (*10 h for IL-6 and TNF) post-sepsis induction. For parameters annotated with ** the significance of differences were analyzed via two-tailed paired t test. For the remaining parameters the significance of differences were analyzed via one-way ANOVA (mixed effect analysis).

	Time after sepsis induction		
Animal analyzed	Baseline	8 h (*10 h)	*p*-value
	*n* = 9	*n* = 9	
**Hemodynamic Parameters**			
**CO (ng/mL)**	4.1 ± 0.9	4.2 ± 1.8	0.8637
**HR (bpm)**	85.6 ± 20.6	161.6 ± 46.5	0.0058
**MAP (mmHg)**	68.4 ± 7.0	59.7 ± 10.8	0.1058
**SVR (dynes·sec/cm⁵)**	1201.7 ± 231.4	1155.9 ± 558.9	0.7841
Respiratory Parameters			
**FiO2 (%)**	32.2 ± 4.4	48.6 ± 19.5	0.0068
**SpO2 (%)**	100.0 ± 0.0	96.6 ± 2.5	0.0093
**SaO2 (%)**	99.5 ± 0.5	93.1 ± 3.8	0.0088
**SvO2 (%)**	60.9 ± 6.9	53.4 ± 13.3	0.2139
**ScvO2 (%)**	62.0 ± 10.0	50.7 ± 13.2	0.0635
**gapCO2 (mmHg)**	7.6 ± 4.8	8.6 ± 3.9	0.6314
Blood and Hematologic Parameters			
**Hb (g/dL)**	8.3 ± 0.5	10.1 ± 1.1	0.0019
**Hct (%)**	25.5 ± 1.6	31.3 ± 3.1	0.0011
Leukocytes (10^9/L)**	13.4 ± 3.0	7.5 ± 11.8	0.164
Platelets (10^9/L)**	271.3 ± 51.9	125.4 ± 72.7	< 0.0001
Biomarkers and Metabolic Parameters			
**DPP3 (U/L)**	517.0 ± 272.5	302.0 ± 89.4	0.0318
**cLac (mmol/L)**	1.2 ± 0.4	2.1 ± 1.2	0.1389
CRP (mg/L)**	5.4 ± 1.2	7.3 ± 2.1	0.1102
IL-6 (pg/mL)*	0.0 ± 0.0	86560.9 ± 74632.7	0.0332
TNF (pg/mL)*	7.0 ± 2.5	324.8 ± 228.6	0.0156
ALP (U/L)**	132.8 ± 49.2	217.8 ± 65.5	0.0519
AST (U/L)**	27.3 ± 5.1	66.3 ± 33.0	0.0742
ALT (U/L)**	42.0 ± 11.9	30.5 ± 10.0	0.0465
GGT (U/L)**	31.8 ± 3.5	41.4 ± 23.2	0.4035
Creatinine (mg/dL)**	96.7 ± 22.8	130.0 ± 58.4	0.2037
Total Bilirubin (mg/dL)**	< 2	9.0 ± 3.5	0.0203
Fluid and Electrolyte Balance			
**EVLW (mL)**	311.0 ± 27.2	376.0 ± 59.7	0.0321
Sodium (mmol/L)**	138.4 ± 1.9	137.8 ± 3.5	0.5281
Potassium (mmol/L)**	4.7 ± 0.3	6.2 ± 0.9	0.0008

**Fig. 3:**
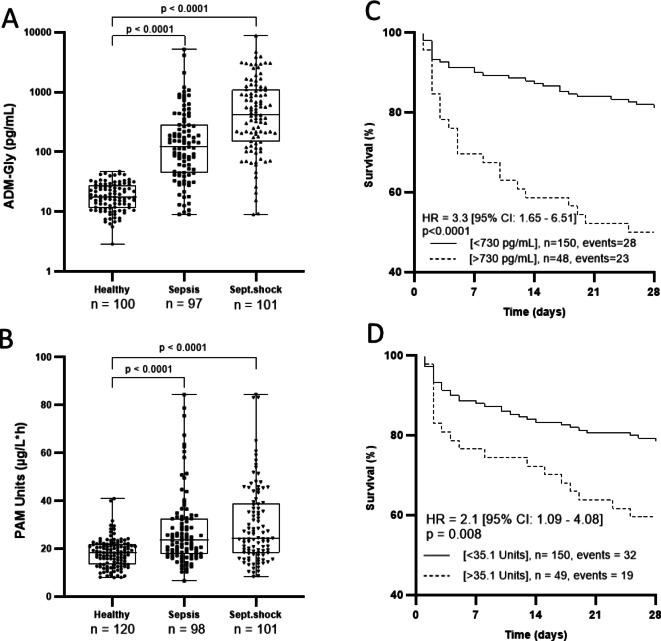
ADM-Gly concentration and PAM activity in AdrenOSS-1 subcohort. Concentration of ADM-Gly (**A**) and PAM activity (**B**) in Li-heparin plasma measured upon admission to the ICU in patients with sepsis and septic shock. The control cohort comprised self-reported healthy individuals. Difference in ADM-Gly concentrations and amidating activity were analyzed via one-way ANOVA (Dunn’s correction). Significance of differences is shown in each panel. Twenty-eight-day Kaplan-Meier survival curves of low versus high ADM-Gly concentrations (**C**) and PAM activity (**D**) based on a cutoff value of 730 pg/mL for ADM-Gly and 35.1 Units for PAM activity in patients with sepsis and septic shock. n is the number of subjects.

To address the alterations along the ADM/PAM axis during the fully manifested stage of sepsis, we analyzed the concentrations of bio-ADM, ADM-Gly and PAM activity in a cohort of patients with sepsis (AdrenOSS-1). The levels of ADM-Gly and PAM activity in Li-heparin plasma were measured upon admission to the ICU. The median concentration of ADM-Gly was significantly elevated in patients with sepsis (121.5 pg/mL [IQR: 44.4–284.1 pg/mL]; *p* < 0.0001) and septic shock (419.3 pg/mL [IQR: 148.8–1106 pg/mL]; *p* < 0.0001), when compared to the self-reported healthy individuals (17.5 pg/mL [IQR: 11.5–27.2 pg/mL]) (Fig. 3A). Similarly, median PAM-AMA levels in sepsis and septic shock were also significantly elevated, with levels of 23.5 Units [IQR: 17.7–32.7 Units; *p* < 0.0001] and 22.4 Units [IQR: 18.2–38.9 Units; *p* < 0.0001], respectively, compared to healthy individuals at 18.4 Units [IQR: 13.5–21.9 Units] (Fig. 3B).

As previously shown for bio-ADM^19^, elevated levels of ADM-Gly and PAM-AMA were associated with higher mortality rates in sepsis and septic shock. Patients with ADM-Gly values lower than 730 pg/mL upon ICU admission had a better survival prognosis in both sepsis and septic shock, with a Mantel-Haenszel hazard ratio (HR) of 0.20 (95% confidence interval (CI): 0.10–0.40; *p* < 0.0001). In contrast, the 28-day mortality for the patients with an ADM-Gly value higher than 730 pg/mL was ca. 50% (Fig. 3C). Similarly, patients with PAM-AMA levels lower than 35.1 Units upon ICU admission had a survival outcome of ca. 80% in sepsis and septic shock, while higher amidating activity led to a mortality rate of ca. 40% (*R* = 0.40 (95% CI: 0.21–0.79; *p* = 0.008); (Fig. 3D). Finally, the median concentration of ADM-Gly (187.6 pg/mL [IQR: 68.7–445.5]) and the median levels of PAM-AMA (23.5 Units [IQR: 17.5–32.7]) in survivors were significantly lower when compared to non-survivors (603.5 pg/mL [IQR: 131.1–1443] for ADM-Gly; 28.3 Units [IQR: 19.0–45.1] for PAM activity) (Fig. S1).

**Fig. 4:**
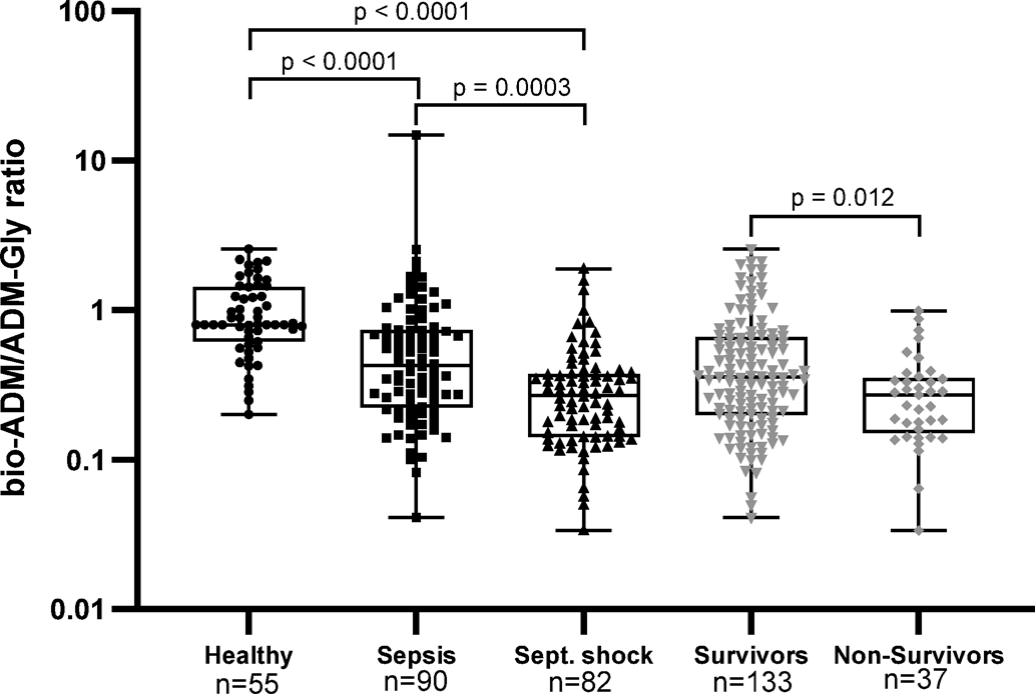
Bio-ADM/ADM-Gly ratio in AdrenOSS-1 cohort. The control cohort comprised self-reported healthy individuals. Differences in bio-ADM/ADM-Gly ratio in sepsis, septic shock and healthy individuals were analyzed via one-way ANOVA (nonparametric Kruskal-Wallis test). The difference in bio-ADM/ADM-Gly ratio between survivors and non-survivors was calculated via unpaired nonparametric Mann-Whitney test. Significance of differences is shown in each panel. n is the number of subjects.

There were no significant differences in ADM-Gly and PAM-AMA levels between patients with sepsis and septic shock. However, the bio-ADM/ADM-Gly ratio differed significantly between groups (unpaired nonparametric Mann-Whitney test, *p* < 0.0003). The lowest median bio-ADM/ADM-Gly ratio of 0.27 [IQR: 0.14–0.38] was observed in septic shock patients, while sepsis patients and healthy individuals had median ratios of 0.43 [IQR: 0.23–0.73] and 0.8 [IQR: 0.61–1.43], respectively (Fig. 4). Additionally, a significantly higher median bio-ADM/ADM-Gly ratio was observed in survivors (0.36 [IQR: 0.20–0.66]) than in non-survivors (0.27 [IQR: 0.15–0.35]; (*p* = 0.012) (Fig. 4).

## Discussion

The present study provides insights into sepsis progression, encompassing the pre-septic, early septic, and post-ICU stages. This is achieved by monitoring the activation kinetics of adrenomedullin, a key regulator of endothelial barrier function and vascular tone, together with its activator PAM enzyme.

Sepsis remains a complex condition with a high mortality rate among hospitalized patients. Early septic state therapy is crucial since prompt intervention can significantly improve survival, prevent organ failure, and reduce the progression to life-threatening septic shock. The Surviving Sepsis Campaign guidelines emphasize the importance of adequate antimicrobial therapy, fluid resuscitation, and ICU admission within the first 3–6 h of diagnosis. Despite advances in sepsis biomarker research, the best combinations for diagnosis, treatment, and outcomes remain unidentified, as was recently reviewed by Barichello et al. Currently, no reliable biomarker can accurately predict sepsis onset, hindering targeted interventions^1^. Bio-ADM is a biomarker that has been the subject of intensive preclinical and clinical research in recent years, both with regard to diagnostic and prognostic significance, and as a therapeutic target. The role of bio-ADM in sepsis is complex. While a vast body of knowledge suggests that high endogenous levels of bio-ADM are associated with impaired outcomes in patients with sepsis and correlate with the severity of pathology, exogenous administration of bio-ADM and/or bio-ADM-stabilizing antibodies improved outcomes in various in vivo animal models (summarized in Tab S2). The latter can be explained by stabilizing bio-ADM in circulation and thus unravelling its protective role on endothelial cell barrier integrity, which is known to be compromised in sepsis, while simultaneously inhibiting its detrimental effect on vascular tone and blood pressure reduction. Therefore, increasing bio-ADM concentration could be a viable therapeutic strategy for sepsis; however, it may be crucial to initiate this elevation earlier in the course of disease progression to facilitate timely endothelial repair.

Clinical data from patients with sepsis or septic shock are not sufficient to provide a comprehensive understanding of the bio-ADM activation pathway during septic onset, as they cannot resolve the critical timeframe until diagnosis. The combination with observations from our porcine sepsis model allows us to gain insight into the early stage of sepsis to describe the hourly-resolved kinetics of bio-ADM activation.

In our porcine sepsis model, both bio-ADM and ADM-Gly levels increased significantly from baseline within the first 2 h after sepsis induction. Notably, the increase in ADM-Gly was significantly higher (27-fold increase compared to the basal concentration) than the 7-fold elevation of bio-ADM concentration within the first 4 h after sepsis induction. This resulted in a significant excess of ADM-Gly over bio-ADM, as reflected by the decrease in the bio-ADM/ADM-Gly ratio relative to the baseline levels. The concentration of ADM-Gly remained significantly elevated over that of bio-ADM throughout the observation period. Compared with the rapid increase in ADM-Gly, an increase in amidating activity occurred with a delay of 6 h after sepsis induction. This temporal delay between the early rise in ADM-Gly and the later rise in PAM activity suggests that amidation of ADM-Gly within secretory vesicles may be incomplete, leading to the predominant release of ADM-Gly into the circulation. In fact, adrenomedullin appears to be stored mainly as ADM-Gly in the secretory granules also under non-pathological conditions^22,26^, further indicating that C-terminal amidation is not fully completed intracellularly. While our data do not clarify whether the subsequent rise in bio-ADM results from intracellular or extracellular amidation, several lines of evidence support the feasibility of C-terminal amidation in circulation^23,27^. A general concern is that extracellular amidation might be biochemically unlikely, since the pH and cofactor conditions in blood differ from the acidic, cofactor-rich environment of secretory granules. However, Kaufmann et al. showed that PAM remains active at physiological pH (~ 7.5)^23^, and Cao et al. demonstrated that exogenous AM-Gly is converted to mature bio-ADM by the rat aortic endothelium in situ, indicating local extracellular amidation by PAM, as this conversion was inhibited by an alpha-amidation enzyme blocker^27^. Together, this suggests that circulating PAM could partially complement incomplete intracellular processing.

Additional support for this concept comes from Kaufmann et al. (2024), who showed in a mild CLP rodent sepsis model that ADM-Gly levels remained significantly elevated compared to bio-ADM up to seven days after sepsis induction, resulting in a persistently low bio-ADM/ADM-Gly ratio. Although PAM activity was not measured in that study, the sustained excess of precursor relative to mature bio-ADM indirectly supports our observation that amidating capacity may be insufficient under ongoing inflammatory stress.

The AdrenOSS-1 human data further suggests that amidation capacity may be a limiting factor in clinical sepsis: ADM-Gly concentration and PAM activity were significantly higher in patients with sepsis and septic shock at the time of ICU admission than in healthy individuals. Similar findings were reported for bio-ADM concentrations in the same cohort by Mebazaa et al. 2018. Given the different observation periods in the subjects from the AdrenOSS-1 study, which reflects manifested sepsis, it is expected that ADM-Gly and PAM activity are higher in these patients than in healthy individuals. Elevated levels of ADM-Gly and PAM were also associated with increased 28-day mortality, indicating their potential as diagnostic biomarkers for sepsis. However, with respect to 28-day mortality, bio-ADM levels were the strongest predictors, with an unadjusted hazard ratio of ~ 2.3 per log unit increase and an adjusted HR of ~ 1.6 when accounting for illness severity. Persistently high bio-ADM over the first 48 h was associated with a nearly fivefold higher mortality risk (HR ~ 4.9). In our study, patients with high ADM-Gly levels (> 730 pg/mL) had a significantly increased risk of 28-day mortality (HR ~ 3.3). Similarly, patients with high PAM activity (> 35.1 Units) also had a significantly increased mortality risk (HR ~ 2.1). Thus, while ADM-Gly and PAM alone show strong prognostic relevance, the magnitude of risk remains comparable to, but generally slightly lower than, that of bio-ADM in AdrenOSS-1. Additionally, the bio-ADM/ADM-Gly ratio was significantly lower in patients with sepsis and septic shock than in healthy individuals, and this lower ratio was associated with increased 28-day mortality. Unlike PAM and ADM-Gly alone, the bio-ADM/ADM-Gly ratio showed significant differences between sepsis and septic shock, suggesting that it could serve as a potential biomarker for both survival outcomes and disease severity. The lower bio-ADM/ADM-Gly ratio in non-survivors compared to survivors suggests insufficient amidation of ADM-Gly, which may impair recovery of the compromised endothelial barrier function mediated by bio-ADM^3,4^. The bio-ADM/ADM-Gly ratio thus adds mechanistic nuance by indicating that a high precursor supply but limited conversion may contribute to more severe septic shock and worse outcomes.

Therapeutically, supplementing PAM to enhance extracellular amidation may represent a novel strategy to improve endothelial protection during early sepsis. Ilina et al. (2025) addressed PAM’s short half-life by PEGylating the enzyme, making it stable and active in plasma^28^. In their preclinical model, a single i.p. dose of PEG-PAM maintained elevated amidating activity in circulation for over a week, supporting its therapeutic potential. Given the early and abundant release of ADM-Gly in sepsis, providing exogenous PAM could accelerate its conversion to bio-ADM, closing the time gap between substrate availability and enzyme activity and thereby enhancing endothelial protection when it is most needed. Since over 70 peptide hormones require amidation, many of which have endothelial protective functions, such as VIP and PACAP, enhancing the active forms of these peptides via PAM supplementation could positively impact endothelial health along with bio-ADM.

One potential limitation of this therapeutic approach is the common vitamin C depletion in septic patients. Patients with severe sepsis often have low vitamin C levels, and although controversial, high-dose vitamin C therapy has been linked to improved outcomes, including reduced inflammation, better organ function, and greater hemodynamic stability. Given that vitamin C is a cofactor for PAM, there may be a causal link between PAM activity and improved patient outcomes, which adds an additional mechanistic perspective to the potential benefit of vitamin C supplementation in sepsis. In the AdrenOSS-1 study, depletion of vitamin C could explain the impaired amidation of ADM-Gly; unfortunately, no data on the vitamin C status of participants were present within the study. One option could be to combine PAM supplementation with vitamin C co-administration to ensure optimal enzymatic activity in vivo. However, while the PAM activity in the AdrenOSS-1 cohort might be compromised, this is not reflected by the determined PAM activity, since the method we use to determine amidating activity is performed under optimal cofactor conditions, reflecting not the endogenous activity (which may be lower than the measured activity in the PAM activity assay), but rather the total amount of PAM in circulation. Therefore, the specific role of PAM in sepsis requires further investigation.

Based on these findings, one possible future approach could be to monitor ADM-Gly and PAM activity levels early in sepsis, together with bio-ADM, which is already under clinical evaluation as a prognostic biomarker. A high ADM-Gly level together with a low bio-ADM/ADM-Gly ratio and low PAM activity may identify patients with insufficient endogenous amidation who could benefit from timely targeted interventions, such as PAM supplementation alone or combined with cofactor support (e.g., vitamin C). However, this concept requires prospective clinical validation to define specific biomarker cut-offs, optimal timing for measurement and intervention, and standardized treatment protocols before it can be developed into a reliable biomarker-guided treatment algorithm.

**Fig. 5:**
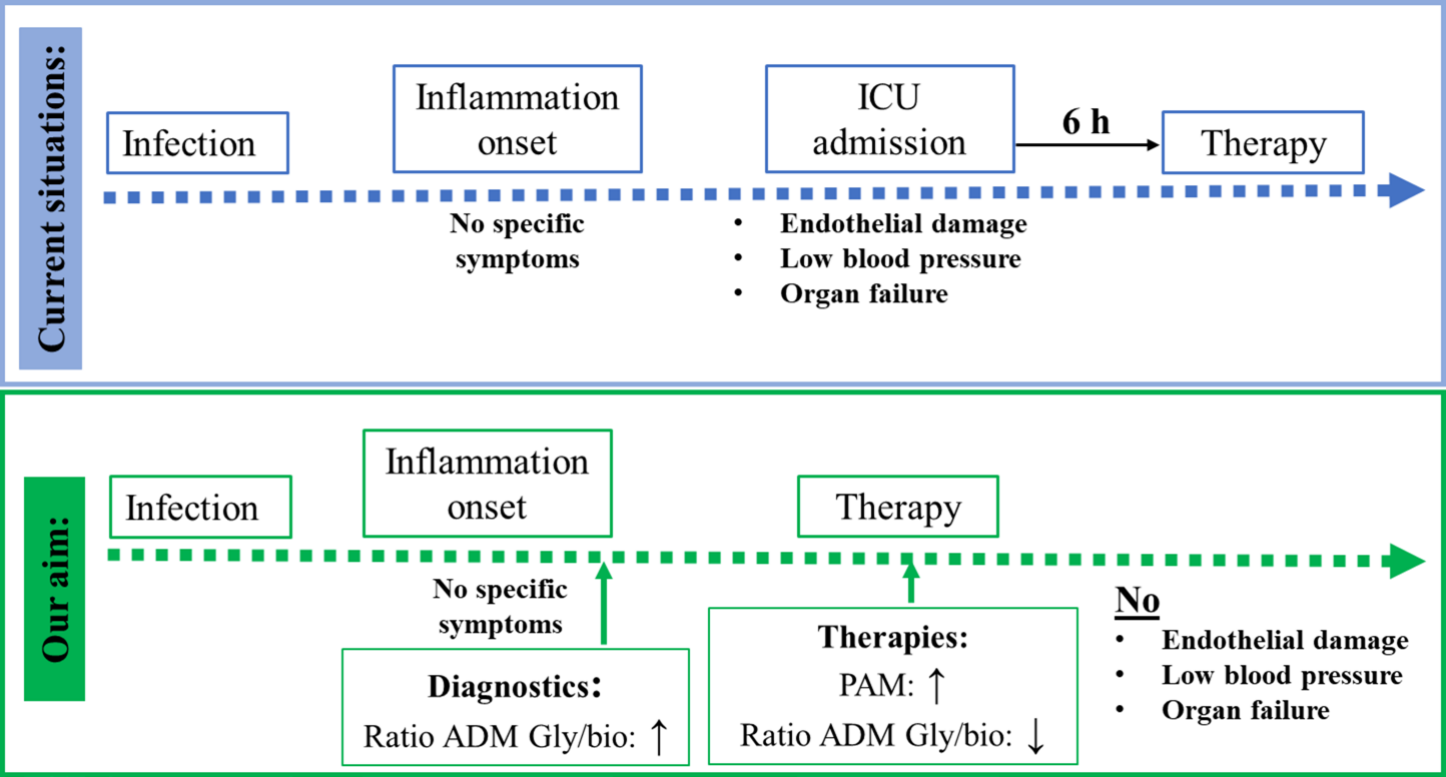
The potential strategy for sepsis therapy, based on the early intervention in the peptide hormone homeostasis.

In summary, the results of our study indicate that in addition to bio-ADM, ADM-Gly and PAM can be suitable biomarkers for sepsis and septic shock, aiding in disease severity stratification and the prediction of 28-day mortality. Notably, ADM-Gly has the added benefit of displaying a stronger concentration increase than bio-ADM. This can enable earlier diagnosis and prompt initiation of appropriate therapy (Fig. 5). Furthermore, these data indicate an active role for circulating PAM in the activation of excess ADM-Gly and possibly other glycine-extended peptide hormones. However, further studies are required to explore the clinical significance and therapeutic applications of ADM-Gly and PAM.

## Conclusion

This study highlights the complex dynamics of adrenomedullin homeostasis in sepsis and suggests that early measurement of ADM-Gly and PAM activity may help refine risk stratification. The significant elevation of ADM-Gly and PAM activity at sepsis onset observed in both porcine models and human patients suggests their potential as early biomarkers. Additionally, the bio-ADM/ADM-Gly ratio is a promising indicator of sepsis severity and correlates with patient survival rates. The differential increase in ADM-Gly compared with bio-ADM, along with the delayed upregulation of PAM activity, raises the possibility that amidation efficiency could become a limiting factor during sepsis progression. In the long term, these insights may help guide biomarker-driven strategies to identify patients with insufficient amidation capacity who could benefit from targeted PAM modulation or cofactor support. Ultimately, this approach could contribute to maintaining endothelial barrier integrity and improving outcomes in septic shock, but prospective clinical validation will be essential.

## Electronic supplementary material

Below is the link to the electronic supplementary material.

Supplementary Material 1

## Data Availability

The datasets used and/or analyzed during the current study are available from the corresponding author upon reasonable request.

## Abbreviations

ADM: adrenomedullin
ADM-Gly: penultimate inactive adrenomedullin precursor with C-terminal glycine
AMA: amidating activity
bio-ADM: active adrenomedullin
cAMP: Cyclic Adenosine Monophosphate
CT-proADM: C-terminal proadrenomedullin fragment
CRLR: calcitonin receptor-like receptor
ICU: intensive care unit
IQR: interquartile range
MR-proADM: mid-regional proadrenomedullin
NO: nitric oxide
PAM: peptidylglycine α-amidating monooxygenase
PAMP: N-terminal 20-peptide
PAL: peptidyl-α-hydroxyglycine α-amidating lyase
PaCO_2_: partial pressure of CO_2_ in arterial blood
PEEP: positive end-expiratory pressure
PHM: peptidylglycine α-hydroxylating monooxygenase
RAMP2: receptor activity-modifying protein 2
RAMP3: receptor activity-modifying protein 3
VSMCs: vascular smooth muscle cells
