# Correction: Kinetics of adrenomedullin pathway activation in a porcine sepsis model and a human cohort of sepsis and septic shock

**DOI:** 10.1038/s41598-025-26642-5

**Published:** 2025-11-04

**Authors:** Christoph Thiele, Yulia Ilina, Paul Kaufmann, Gero Springsfeld, Michaela Press, Oliver Hartmann, Andreas Bergmann, Thomas Breuer, Gernot Marx, Tim-Philip Simon

**Affiliations:** 1 Department of Intensive Care and Intermediate Care, University Hospital RWTH Aachen, Pauwelsstr. 30, 52074 Aachen, Germany; 2 PAM Theragnostics GmbH, Neuendorfstr. 15A, 16761 Hennigsdorf, Germany; 3 Sphingotec GmbH, 16761 Hennigsdorf, Germany

Correction to: *Scientific Reports* 10.1038/s41598-025-19278-y, published online 24 September 2025

The original version of this Article contained errors in the names of the authors Christoph Thiele, Yulia Ilina, Paul Kaufmann, Gero Springsfeld, Michaela Press, Oliver Hartmann, Andreas Bergmann, Thomas Breuer, Gernot Marx, Tim-Philip Simon which were incorrectly given as Thiele Christoph, Ilina Yulia, Kaufmann Paul, Springsfeld Gero, Press Michaela, Hartmann Oliver, Bergmann Andreas, Breuer Thomas, Marx Gernot & Simon Tim-Philip.

The original Article has been corrected.

