# Supplementary material for: Kinetics of adrenomedullin pathway activation in a porcine sepsis model and a human cohort of sepsis and septic shock
Source: Sci Rep. 2025 Sep 24;15:32693. doi: 10.1038/s41598-025-19278-y (PMC12460767; doi:10.1038/s41598-025-19278-y)
Supplement: Supplementary file 1 — Supplementary Material 1 [file 41598_2025_19278_MOESM1_ESM.docx]

**Supplementary**

*
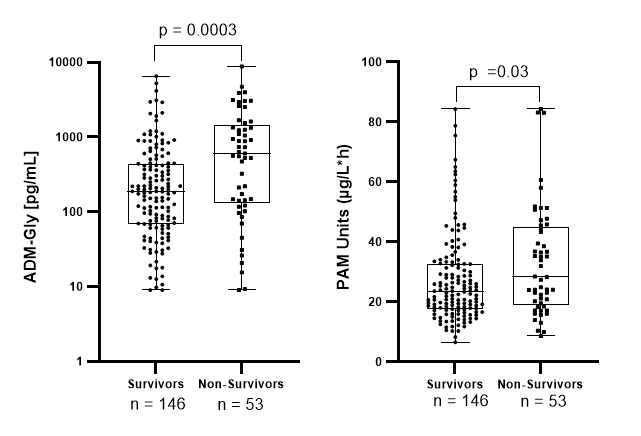
*

***Figure S1****. Comparative analysis of ADM-Gly concentration and PAM activity in survavors and non-survivors in AdrenOSS-1 subcohort (non-parametric Mann-Whitney test). Significance of differences (p value) is shown in each panel. n is the number of subjects.*

*Table S1: Comprehensive time course of physiological and biochemical parameters, including hemodynamic parameters, respiratory parameters, blood and hematologic parameters, biomarkers and metabolic parameters, and fluid and electrolyte balance in porcine sepsis-induced model. DPP3 (Dipeptidyl Peptidase 3), CO (Cardiac Output), HR (Heart Rate), MAP (Mean Arterial Pressure), SVR (Systemic Vascular Resistance), EVLW (Extravascular Lung Water), gapCO2 (Carbon Dioxide Gap), mPAP (Mean Pulmonary Artery Pressure), SvO2 (Mixed Venous Oxygen Saturation), Hb (Hemoglobin), SaO2 (Arterial Oxygen Saturation), Hct (Hematocrit), cLac (Lactate Concentration), ScvO2 (Central Venous Oxygen Saturation), FiO2 (Fraction of Inspired Oxygen), SpO2 (Peripheral Oxygen Saturation), ALP (Alkaline Phosphatase), AST (Aspartate Aminotransferase), ALT (Alanine Aminotransferase), GGT (Gamma-Glutamyl Transferase), CRP (C-Reactive Protein), IL-6 (Interleukin 6), TNF (Tumor Necrosis Factor). The values are presented as mean ± standard deviation with n being the number of animals in each group, at baseline (BL), 2h, 4h, 6h, 8h, 10h, 12h and 14h post-sepsis induction. n.d. stands for not determined.*

|  |  | **Time after sepsis induction** | | | | | | |
| --- | --- | --- | --- | --- | --- | --- | --- | --- |
|  | **BL** | **2h** | **4h** | **6h** | **8h** | **10h** | **12h** | **14h** |
| **Animal analyzed** | n = 9 | n = 9 | n = 9 | n = 9 | n = 9 | n = 7 | n = 6 | n = 3 |
| Hemodynamic Parameters | | | | | | | | |
| **CO (ng/mL)** | 4.1 ± 0.9 | 4.1 ± 0.8 | 3.2 ± 0.8 | 3.7 ± 1.1 | 4.2 ± 1.8 | 5.4 ± 1.4 | 5.6 ± 0.9 | 7.2 ± 1.0 |
| **HR (bpm)** | 85.6 ± 20.6 | 119.1 ± 37.9 | 172.9 ± 25.0 | 167.4 ± 32.5 | 161.6 ± 46.5 | 155.6 ± 27.4 | 155.0 ± 43.6 | 178.0 ± 17.1 |
| **MAP (mmHg)** | 68.4 ± 7.0 | 68.2 ± 10.3 | 64.9 ± 13.7 | 64.7 ± 10.5 | 59.7 ± 10.8 | 58.6 ± 4.6 | 57.8 ± 6.6 | 59.3 ± 8.1 |
| **SVR (dynes·sec/cm⁵)** | 1201.7 ± 231.4 | 1233.9 ± 314.7 | 1555.8 ± 438.7 | 1362.2 ± 587.3 | 1155.9 ± 558.9 | 865.5 ± 169.8 | 703.3 ± 53.9 | 563.3 ± 75.9 |
| Respiratory Parameters | | | | | | | | |
| **FiO2 (%)** | 32.2 ± 4.4 | 32.2 ± 4.4 | 38.9 ± 6.0 | 51.1 ± 25.2 | 48.6 ± 19.5 | 60.0 ± 30.0 | 61.7 ± 29.9 | n.d. |
| **SpO2 (%)** | 100.0 ± 0.0 | 98.9 ± 2.4 | 96.3 ± 2.8 | n.d. | 96.6 ± 2.5 | 93.0 ± 4.5 | 93.8 ± 5.6 | n.d. |
| **SaO2 (%)** | 99.5 ± 0.5 | 97.2 ± 1.8 | 93.2 ± 4.0 | 92.6 ± 5.1 | 93.1 ± 3.8 | 90.7 ± 8.2 | 86.6 ± 16.3 | 91.7 ± 4.5 |
| **SvO2 (%)** | 60.9 ± 6.9 | 59.0 ± 8.0 | 42.9 ± 12.5 | 51.4 ± 20.8 | 53.4 ± 13.3 | 53.3 ± 5.3 | 51.2 ± 20.9 | 65.0 ± 5.6 |
| **ScvO2 (%)** | 62.0 ± 10.0 | 58.9 ± 4.6 | 43.3 ± 11.3 | 46.3 ± 14.8 | 50.7 ± 13.2 | 53.8 ± 23.3 | 49.2 ± 18.8 | 68.6 ± 11.6 |
| **gapCO2 (mmHg)** | 7.6 ± 4.8 | 9.3 ± 3.7 | 13.7 ± 8.4 | 13.0 ± 4.8 | 8.6 ± 3.9 | 9.0 ± 3.9 | 10.3 ± 7.5 | n.d. |
| Blood and Hematologic Parameters | | | | | | | | |
| **Hb (g/dL)** | 8.3 ± 0.5 | 10.8 ± 0.8 | 11.9 ± 1.0 | 11.3 ± 0.8 | 10.1 ± 1.1 | 8.8 ± 0.8 | 8.2 ± 0.5 | 7.8 ± 0.5 |
| **Hct (%)** | 25.5 ± 1.6 | 33.2 ± 2.5 | 36.6 ± 3.0 | 34.8 ± 2.5 | 31.3 ± 3.1 | 27.4 ± 2.4 | 25.3 ± 1.6 | 24.2 ± 1.5 |
| **Leukocytes (10^9/L)** | 13.4 ± 3.0 | n.d. | n.d. | n.d. | 7.5 ± 11.8 | n.d. | n.d. | 02. Sep |
| **Platelets (10^9/L)** | 271.3 ± 51.9 | n.d. | n.d. | n.d. | 125.4 ± 72.7 | n.d. | n.d. | 70 |
| Biomarkers and Metabolic Parameters | | | | | | | | |
| **DPP3 (U/L)** | 517.0 ± 272.5 | 491.8 ± 121.6 | 329.5 ± 84.7 | 342.8 ± 79.9 | 302.0 ± 89.4 | 302.4 ± 99.8 | 285.2 ± 79.5 | 290.1 ± 119.8 |
| **Bio-ADM (pg/mL)** | 8.8 ± 1.3 | 28.0 ± 12.9 | 63.4 ± 20.5 | 130.4 ± 67.9 | 183.5 ± 120.5 | 314.5 ± 165.6 | 333.3 ± 110.1 | 379.8 ± 200.0 |
| **ADM-Gly (pg/mL)** | 26.5 ± 11.2 | 260.8 ± 92.0 | 728.7 ± 378.3 | 869.0 ± 346.6 | 824.3 ± 390.5 | 989.4 ± 431.3 | 1061.0 ± 494.7 | 964.9 ± 639.3 |
| **Bio-ADM/ADM-Gly** | 0.37 ± 0.12 | 0.11 ± 0.03 | 0.10 ± 0.03 | 0.16 ± 0.05 | 0.23 ± 0.11 | 0.33 ± 0.12 | 0.34 ± 0.08 | 0.45 ± 0.17 |
| **PAM (Units)** | 23.4 ± 3.9 | 23.3 ± 5.8 | 32.2 ± 13.9 | 39.3 ± 10.5 | 47.0 ± 9.5 | 56.3 ± 12.9 | 60.7 ± 8.5 | 59.6 ± 15.8 |
| **cLac (mmol/L)** | 1.2 ± 0.4 | 1.6 ± 0.6 | 2.9 ± 1.4 | 2.3 ± 1.2 | 2.1 ± 1.2 | 2.9 ± 2.0 | 4.1 ± 3.0 | 3.9 ± 4.0 |
| **CRP (mg/L)** | 5.4 ± 1.2 | n.d. | n.d. | n.d. | 7.3 ± 2.1 | n.d. | n.d. | 6 |
| **IL-6 (pg/mL)** | 0.0 ± 0.0 | 3560.7 ± 3624.7 | 70293.6 ± 66509.5 | 81754.5 ± 72782.8 | n.d. | 86560.9 ± 74632.7 | n.d. | n.d. |
| **TNF (pg/mL)** | 7.0 ± 2.5 | 157557.5 ± 146477.1 | 7053.0 ± 8058.5 | 1496.3 ± 3264.6 | n.d. | 324.8 ± 228.6 | n.d. | n.d. |
| **ALP (U/L)** | 132.8 ± 49.2 | n.d. | n.d. | n.d. | 217.8 ± 65.5 | n.d. | n.d. | 328 |
| **AST (U/L)** | 27.3 ± 5.1 | n.d. | n.d. | n.d. | 66.3 ± 33.0 | n.d. | n.d. | 171 |
| **ALT (U/L)** | 42.0 ± 11.9 | n.d. | n.d. | n.d. | 30.5 ± 10.0 | n.d. | n.d. | 38 |
| **GGT (U/L)** | 31.8 ± 3.5 | n.d. | n.d. | n.d. | 41.4 ± 23.2 | n.d. | n.d. | 31 |
| **Creatinine (mg/dL)** | 96.7 ± 22.8 | n.d. | n.d. | n.d. | 130.0 ± 58.4 | n.d. | n.d. | 141 |
| **Total Bilirubin (mg/dL)** | < 2 | n.d. | n.d. | n.d. | 9.0 ± 3.5 | n.d. | n.d. | 6 |
| Fluid and Electrolyte Balance | | | | | | | | |
| **EVLW (mL)** | 311.0 ± 27.2 | 311.9 ± 51.9 | 330.4 ± 58.5 | 347.0 ± 91.8 | 376.0 ± 59.7 | 426.5 ± 69.8 | 427.0 ± 90.7 | 513.0 ± 92.7 |
| **Sodium (mmol/L)** | 138.4 ± 1.9 | n.d. | n.d. | n.d. | 137.8 ± 3.5 | n.d. | n.d. | 135 |
| **Potassium (mmol/L)** | 4.7 ± 0.3 | n.d. | n.d. | n.d. | 6.2 ± 0.9 | n.d. | n.d. | 8.5 |

*Table S2. Overview of in vivo studies demonstrating that exogenous or stabilized adrenomedullin (ADM) reduces sepsis severity and improves survival*

| **ADM type used** | **Sepsis model** | **Main findings** | **Reference** |
| --- | --- | --- | --- |
| Exogenous rat ADM | *S. aureus* α-toxin-induced shock (rat) | Reduced mortality (53% → 7%) and vascular leakage | Temmesfeld-Wollbrück et al., 2007; DOI: 10.1007/s00134-007-0561-y |
| Human ADM + AMBP-1 | Intestinal ischemia/reperfusion (rat) | Co-administration reduced pro-inflammatory cytokines, attenuated organ injury, and improved survival in a dose-dependent manner | Yang et al., 2009; DOI: 10.1097/SLA.0b013e3181961d43 |
| Human ADM + AMBP-1 | Obstructive jaundice (bile duct ligation) + cecal ligation and puncture (CLP) in rats | Human ADM/AMBP-1 treatment reduced tissue injury, dampened inflammation, and improved 7-day survival from 21–53% in jaundiced rats with polymicrobial sepsis. | Yang et al., 2010; DOI: 10.1016/j.peptides.2010.01.010 |
| Exogenous human ADM | HUVECs and porcine pulmonary artery endothelial cells exposed to H₂O₂, thrombin, and E. coli hemolysin (HlyA) in vitro; Isolated perfused rabbit lungs exposed to H₂O₂ (in vivo) | ADM reduced endothelial hyperpermeability by inhibiting MLC phosphorylation and stress fiber formation via cAMP elevation; protected against H₂O₂-induced lung edema | Hippenstiel et al., 2002; DOI: 10.1161/01.RES.0000036603.61868.F9 |
| Endogenous ADM (AM+/– mice with reduced ADM levels) | LPS-induced endotoxemia (mouse) | Mice with reduced ADM expression showed significantly worsened survival, elevated inflammatory cytokine levels, and enhanced vascular leakage, demonstrating a protective role of ADM in vivo. | Nikitenko et al., 2013; DOI: 10.1038/jid.2013.47 |
| Adrecizumab (non-neutralizing ADM-stabilizing Ab) | Rodent models (rats and mice) subjected to LPS-induced endotoxemia and CLP | Adrecizumab administration led to improved vascular barrier function, reduced vascular leakage, and decreased mortality in sepsis models. The antibody increased plasma ADM levels, enhancing endothelial barrier stabilization without causing hypotension. | Geven et al., 2018; DOI: 10.1097/SHK.0000000000001102 |
|  | Porcine two-hit model involving hemorrhagic shock followed by E. coli-induced peritonitis | Adrecizumab treatment resulted in improved hemodynamic stability, reduced fluid requirements, and decreased renal granulocyte infiltration. The antibody increased plasma ADM levels, contributing to vascular barrier protection without adverse effects on blood pressure. | Thiele et al., 2020; DOI: 10.1097/SHK.0000000000001587 |
| Exogenous human ADM | Ovine endotoxaemia (Salmonella typhosa LPS) | ADM prevented and reversed hypodynamic circulation and pulmonary hypertension, and reduced lactate levels | Ertmer et al., 2007; DOI: 10.1093/bja/aem295 |
